# The Positive and Negative Experiences of Caregiving for Siblings of Young People with First Episode Psychosis

**DOI:** 10.3389/fpsyg.2017.00730

**Published:** 2017-05-23

**Authors:** Siann Bowman, Mario Alvarez-Jimenez, Darryl Wade, Linsey Howie, Patrick McGorry

**Affiliations:** ^1^Department of Occupational Therapy, School of Allied Health, La Trobe University, MelbourneVIC, Australia; ^2^The National Centre of Excellence in Youth Mental Health, Orygen, The University of Melbourne, MelbourneVIC, Australia; ^3^The Australia Centre for Post Traumatic Mental Health, The University of Melbourne, MelbourneVIC, Australia; ^4^Department of Occupational Therapy, School of Clinical and Community Allied Health, La Trobe University, MelbourneVIC, Australia

**Keywords:** first episode psychosis, siblings, experience of caregiving

## Abstract

**Background:** The impact of first episode psychosis (FEP) upon parents’ experience of caregiving has been well-documented. However, the determinants and nature of this remain poorly understood in siblings. It is hypothesized that siblings of young people with FEP are also impacted by caregiving and burden. This study aimed to characterize the experience of caregiving for siblings of young people with FEP.

**Method:** Survey methodology was used to explore the experience of 157 siblings in the first 18 months of their brother or sister’s treatment for FEP. Participants reported on their appraisal of the negative and positive aspects of caregiving as measured by the Experience of Caregiving Inventory (ECI). Descriptive statistics were used to establish the results for the total sample as well as for gender and birth order differences. A series of multivariate regression analyses were conducted to determine the relationships between illness characteristics and siblings’ experience of caregiving.

**Results:** Older brothers reported the lowest scores for negative experiences in caregiving and younger sisters reported the highest. Negative experiences in caregiving resulted in less warmth within the sibling relationship and impacted negatively upon quality of life. When the young person with FEP had attempted suicide and/or been physically violent, siblings experienced more caregiver burden. Multivariate analysis showed that female gender was a significant factor in explaining the impact of illness related variables on the experience of caregiving.

**Conclusion:** Suicide attempts and a history of violence resulted in higher caregiving burden for siblings regardless of whether they lived with the young person experiencing FEP or not. Female siblings are at higher risk of negative experiences from caregiving resulting in a reduced quality of life and a changed sibling relationship. Suicide attempts and violence are indicators for intensive case management to improve outcomes for the individual with FEP which may in turn reduce the burden experienced by the sibling. Clinicians can use these findings to identify siblings, assertively intervene and provide increased psychological support, psychoeducation and practical problem solving to reduce the burden. The caregiving role that they already play for their ill brother or sister should be recognized.

## Introduction

The sibling relationship plays a critical, formative and protective role during adolescence and early adulthood (Branje et al., 2004; Milevsky, 2005; Gass et al., 2007). Disruption to the relationship during this stage of life can have significant implications, specifically to personality development, identity formation, and social support. Developmental theories provide a framework for understanding the importance of the sibling relationship and the potential losses and negative impact that may occur with the onset of first episode psychosis (FEP) (Adler, 1928; Festinger, 1954; Bowlby, 1969; Weiss, 1974; Bandura, 1989; Kreppner and Lerner, 1989). Studies show that most young people experiencing FEP live with their parents and siblings (76–88%) therefore siblings experience similar events during prodrome and acute onset as their parents (Gleeson et al., 2008; Lobban and Barrowclough, 2009). Research shows that siblings have more intimate knowledge about their ill brother and sister than parents (Dyregrov and Dyregrov, 2005).

Family research and clinical practice in FEP has neglected siblings (Bowman et al., 2013). International Clinical Guidelines for Early Psychosis (Royal Australian and New Zealand College of Psychiatrists [RANZCP], 2005) recommend family focused interventions to support all members in their role. There is a large body of literature that shows that family interventions do not specifically include siblings and parents are the main participants in research (Tennakoon et al., 2000; Addington et al., 2005; Wong et al., 2009; Gleeson et al., 2010; McCann et al., 2011). Sibling relationships remain neglected in intervention studies (Smith et al., 2009).

The appraisal of caregiving, also known as burden, has been used in studies largely with parents of young people experiencing FEP but again not with siblings (Addington et al., 2005; Patterson et al., 2005; Gleeson et al., 2008, 2010; Alvarez-Jimenez et al., 2010; Boydell et al., 2014; Jansen et al., 2014; Tomlinson et al., 2014). Parents appraise their caregiving experience negatively and face challenges in a number of ways: trying to understand what is happening for their child; negotiating the service system to successfully receive effective help; managing odd, confronting and at times frightening and embarrassing behaviors by their ill child; dealing with uncertain diagnoses and treatment options; adjusting to a new caregiver role; managing the stigma of having a child with a psychotic disorder which can lead to social isolation and loneliness; experience grief and loss for the previous relationship with their child and the plans for the future; experience guilt and blame for thinking they failed to help early enough or that they played a role in causing the illness; and experience changed family routines (Addington et al., 2005; Patterson et al., 2005; Gleeson et al., 2008, 2010; Alvarez-Jimenez et al., 2010; Boydell et al., 2014; Jansen et al., 2014; Tomlinson et al., 2014). There is no previous study investigating the appraisal of caregiving experiences for siblings of young people with FEP.

Research with siblings of individuals with long term psychotic illness identifies commonly occurring issues such as stigma, fear, guilt, grief, changed life goals, changed relationships, worry about the future, objective and subjective burden of care (Titelman and Psyk, 1991; Gerace et al., 1993; Lively et al., 1994; Lukens et al., 2004; Barak and Solomon, 2005; Patterson et al., 2005; Barnable et al., 2006; Friedrich et al., 2008; Smith and Greenberg, 2008; Lobban and Barrowclough, 2009; Ewertzon et al., 2012; Sjoblom et al., 2013; Felton, 2014). These experiences result in a decreased quality of life (Smith and Greenberg, 2008). As a result, the quality of the relationship deteriorates and the positive contributions it can make to both individuals are lost (Barak and Solomon, 2005; Smith and Greenberg, 2008; Felton, 2014). Over 80% of participants in this research are female siblings (Titelman and Psyk, 1991; Gerace et al., 1993; Lively et al., 1994; Lukens et al., 2004; Barak and Solomon, 2005; Patterson et al., 2005; Barnable et al., 2006; Smith and Greenberg, 2008; Lobban and Barrowclough, 2009; Ewertzon et al., 2012; Sjoblom et al., 2013; Felton, 2014).

It is difficult to conceive that family work can occur without the involvement of siblings both in terms of the unique importance of the relationship and the growing literature documenting the pervasive impact of FEP on sibling lives (Newman et al., 2011; Sin et al., 2011, 2013; Bowman et al., 2013, 2014, 2015). Qualitative studies in the United Kingdom with siblings of individuals with FEP show that common experiences include resentment, grief, loss, blame, stigma, fear of becoming ill themselves, shame, powerlessness, stop inviting friends home, keep their ill brother or sister’s secrets, provide support to their parents rather than the other way around, and provide a great deal of direct and indirect care within the family (Sin et al., 2008, 2011, 2013). Sisters have been found to have difference experiences to brothers (Newman et al., 2011; Sin et al., 2011; Bowman et al., 2014, 2015).

Even though FEP intervention promotes optimism for a good outcome with evidence based care to achieve this, the literature indicates that many siblings will experience their brother or sister requiring hospital admissions, being non-compliant with treatment, having persistent psychotic symptoms, engaging in ongoing substance use, attempting suicide and/or being physical violent (Coldham et al., 2002; Nordentoft et al., 2002; Power et al., 2003; Wade et al., 2004, 2006; Addington and Addington, 2007; Robinson et al., 2009; Farrelly et al., 2010; Lambert et al., 2010). The evidence also suggests that individuals can find it hard to access treatment and care and resist obtaining help. This can lead to long periods of untreated psychosis, which can impact upon the prospects of recovery (Perkins et al., 2005). Australian studies have shown that when the young person with FEP has attempted suicide and/or has a history of violence, there is a significant and negative impact on the sibling relationship and their quality of life (Bowman et al., 2013, 2014, 2015).

This study endeavors to discover what the experience of caregiving is like for siblings of young people with FEP and whether the experience has an impact upon this important relationship and their quality of life. The experience of caregiving may be different depending on gender and birth order and consequently this study also seeks to discover if this is true. The appraisal of caregiving and its relationship to illness related variables is also explored.

## Materials and Methods

### Sample

Siblings of individuals with FEP attending the Early Psychosis Prevention and Intervention Centre (EPPIC), Orygen Youth Health, Melbourne, Australia, were invited to participate in the study. The age range of the EPPIC program at the time of the study was 15–29 years. The mean age for those experiencing FEP was 21.45 (*SD*: 3.51) whilst the mean age for the sibling participant was 21.76 (*SD*: 4.38). Birth order as opposed to age difference was used in the analysis as previous literature shows that birth order has a more significant impact upon development than age difference (Adler, 1928; Kinsella et al., 1996; East, 2009).

The sample included siblings who were fluent in English and able to give informed consent. It was also an ethical requirement to obtain consent from the young person experiencing FEP for their sibling to participate in the study. The exclusion criteria were intellectual impairment and a history of psychosis. The research and ethics committees of the North West Mental Health Program and the La Trobe University approved the study.

There were 388 patients attending EPPIC at the time of recruitment. Sixty-four were lost to follow up, 24 did not have a sibling, and 85 were reported by their case manager or doctor to be too unwell to be approached. Of the remaining 215 young people with FEP, only 123 (57%) agreed to meet with the researcher about gaining consent for their sibling to participate. All 123 patients provided consent for specifically identified siblings to participate. Nine young people with FEP agreed for more than one sibling to participate (seven consented for 2 siblings, one consented to 3 siblings, and one consented to 6 siblings to participate). The age range of the sibling participants was from 17 to 29 years old. Twenty-nine were under 18 years of age and required parental consent (24%). Young people with FEP were not asked why consent was not given for other siblings within the family to participate. It is therefore not known what the differences were between siblings who were refused consent to those who were granted consent in terms of birth order, gender or whether they lived at home.

According to the medical records, the 123 young people with FEP had a total of 417 potential siblings who were eligible to participate in the study however, only 157 (37.6%) were approached because the young person with FEP only provided consent for them to participate (37.6%). The mean number of children in each family (*n* = 123) was 3.32 children with a range of 2–7 children. Further, 39.5% of sibling participants only had one sibling who was the young person experiencing FEP; 31.2% had two siblings (one with FEP), 11.5% had three siblings (one with FEP) and 10% had six siblings (one with FEP). This process resulted in a purposive sample of 157 siblings (see **Tables 1**, **2**).

**Table 1:** Descriptive summary of sibling participant, characteristics as reported by them.

Characteristics (*n* = 157)	Study sample	
	*n*	(%)
Male	81	(51.6)
First born	65	(41.4)
Second born	52	(33.1)
Biological sibling	154	(98.1)
Living with ill brother or sister	99	(63.1)
Employed full time	91	(58)
Competed year 12	115	(73.2)
Completed a tertiary degree	15	(10)
Older brother to person with early psychosis	42	(26.7)
Younger brother to person with early psychosis	39	(24.8)
Older sister to person with early psychosis	44	(28)
Younger sister to person with early psychosis	32	(20.3)
Parents divorced	69	(43.9)
Moved out of home due to their brother or sisters illness	8	(5.1)
Brother or sister required admission due to illness	123	(78.3)
Visited their brother or sister when in hospital	104	(66.2)
Believed their brother or sister remained psychotic	88	(56.1)
Length of duration of untreated psychosis as stated by sibling		
1–6 months	42	(26.7)
7–12 months	61	(38.8)
≥13 months	54	(34.3)
Attended family services offered by mental health clinic	6	(3.8)
Happy with treatment brother or sister receives	113	(72)
Any contact with brother or sisters treating team	27	(17.2)

*The mean age of the sample was 21.7 years (SD = 4.4).*

**Table 2:** Sociodemographic characteristics of young people with early psychosis obtained from medical record (*n* = 123).

Characteristics	Study sample	
	*N*	(%)
Male	87	(70.7)
First born	41	(33)
Second born	56	(45.5)
Employed full time	33	(26.8)
Unemployed	71	(57.7)
At school or university	19	(15.4)
Diagnosis		
Schizophrenia	51	(41.5)
Schizophreniform	47	(38.2)
Schizoaffective	6	(4.9)
Bipolar affective disorder	13	(10.6)
Post traumatic stress disorder	5	(4.1)
Post partum psychosis	1	(0.8)
Persisting psychosis	64	(52)
Length of time in treatment		
1–6 months	47	(38.2)
7–12 months	54	(43.9)
13–18 months	20	(16.2)
>18 month	2	(1.6)
Number of admissions		
No admission	29	(23.6)
1 admission	40	(32.5)
2 admissions	29	(23.6)
=/>3 admissions	25	(20.3)
Compliant with medication	98	(79)
Attempted suicide	45	(36.5)
Completed homicide	2	(1.6)
Past history of substance use	96	(78)
Current substance use	34	(27.6)
Australian nationality	76	(61.7)

*The mean age of young people with early psychosis was 21.4 years (SD = 3.5)2.*

### Measures

It was important for the study to select scales that were reliable and had good internal consistency. Internal consistency refers to the degree to which the items that make up the scale effectively measure the variable. One of the most commonly used indicators of internal consistency is Cronbach’s alpha coefficient. Pallant (2016) reports that ideally the Cronbach alpha coefficient of a scale should be above 0.7. A procedure for checking the reliability of each scale selected for this study was implemented. The overall Cronbach alpha coefficient is therefore reported for each scale to show its internal consistency. The Cronbach alpha coefficient for each subscale is further reported in **Table 8**.

Subjective appraisal of caregiving was measured by the Experience of Caregiving Inventory (ECI) (Szmukler et al., 1996). The ECI is a 66 item self-report measure developed with caregivers (9% were siblings) to assess the subjective negative and positive experiences of caregiving for family members who have a relative with a serious mental illness. Negative caregiving appraisal is calculated from the sum of the eight negative ECI subscales [difficult behaviors, negative symptoms, stigma, problems with services, effects on family, needing to provide backup support (e.g., lending money), dependency and loss], and positive appraisal from the sum of the two positive ECI subscales (rewarding personal experiences; good aspects of relationship with the patient). As this assessment was developed in conjunction with mostly parents and spouses (91%) questions are targeted to these roles and may not specifically address sibling needs. It has however, been used previously with siblings in FEP (Sin et al., 2016). Items are scored based on how often the individual has thought about various statements (e.g., ‘During the past month how often have you thought about feeling unable to tell anyone about the illness?’). The individual rates these statements from 0 (never) to 4 (nearly always). Scores are calculated by adding responses within each subscale and dividing this score by the total number of items to obtain a simple mean. A high rating on the negative subscale reflects a high level of negative experiences. A high rating on the positive subscale reflects a high level of positive experiences. The maximum score for the negative subscale is 208 and 56 for the positive subscale. The internal consistency and construct validity of the ECI has been found to be high and a strong predictor of the psychological well-being for people who have a family member with psychosis (Szmukler et al., 1996).

The Adult Sibling Relationship Questionnaire (ASRQ) (Stocker et al., 1997) was utilized to examine the quality of the sibling relationship. This measure consists of 81 items on 14 scales that combine to form three higher-order factors: warmth (intimacy, affection, knowledge, acceptance, similarity, admiration, emotional support, and instrumental support), conflict (dominance, competition, antagonism, quarreling), and rivalry (maternal rivalry and paternal rivalry). For all ASRQ items, participants rated how characteristic each item is of themselves and their sibling. Participants rate how often feelings and behaviors occur on a Likert Scale from 1 to 5 ranging from (1) hardly at all to (5) to extremely much.

The World Health Organisation Quality of Life Scale – Bref (WHOQoL-Bref) (World Health Organisation [WHO], 1998) was used to assess quality of life. This scale is a 26 item self-report measure organized into four domains: Physical QoL (seven items: activities of daily living and energy levels); Psychological QoL (eight items: self-esteem and negative feelings); Social QoL (three items: personal relationships and social supports); and Environment QoL (eight items: financial resources and home environment). All items are rated on a five-point scale assessing intensity, capacity, frequency, or evaluation of their satisfaction. The mean scores of items within each domain are used to calculate the domain score. Mean scores are then multiplied by four to make domain scores compatible with the scores used in the WHOQOL-100 (scale range: 0–100). This scale required reverse scoring of items 3, 4, and 26. The method for converting raw scores to transformed scores is provided in the WHOQOL-Bref user manual [99]. Domain scores are scaled in a positive direction with higher scores denoting higher quality of life (World Health Organisation [WHO], 1998).

Each client’s medical file was reviewed to obtain data on the duration of untreated psychosis (DUP) before receiving treatment (in months), the number of hospital admissions, history of medication compliance (non-compliant, fluctuating compliance, full compliance), persisting positive symptoms (yes/no), suicide attempts (yes/no; number of), substance use (yes/no; frequency), and history of physical violence (yes/no).

### Data Analyses

Descriptive statistics for demographic and clinical variables were computed. Means, standard deviations (SD), and 95% confidence intervals (95% CI) were calculated to represent the preliminary population norms. Statistical differences between groups were investigated using independent samples *t*-tests and one way analysis of variance (ANOVA). *Post hoc* comparisons were implemented to ascertain which groups were significantly different from one another. Given the exploratory nature of the research, alpha was set at 0.05 for all analyses. No adjustments were made for multiple comparisons as they can result in a higher type II error, reduced power, and increased likelihood of missing significant findings.

Correlation analyses were used to detect relationships between the scale and variables. Pearson’s product-moment co-efficient (*r*) was used. The significance of the strength of correlation was interpreted in accordance with guidelines proposed by Cohen. In order to determine the strength of the relationships, Cohen’s guidelines were used: *r* = 0.10 to 0.29 or *r* = -0.10 to -0.29 small/weak; *r* = 0.30 to 4.9 or *r* = -0.30 to -4.9 moderate; *r* = 0.50 to 1.0 or -0.50 to -1.0 large/strong.

A series of regression analyses were conducted to determine which illness related variables were independent predictors of the negative and positive aspects of care. These variables were entered into two separate regression analyses, with the Negative Aspects of Caregiving Subscale and the Positive Aspects of Caregiving Subscale serving as the dependent variable in each model. All statistical analyses were undertaken using SPSS-24.

## Results

### The Experience of Caregiving, Sibling Gender, and Birth order

Siblings were divided into four groups: older brother (26.8%), younger brother (24.8%), older sister (28%), and younger sister (20.4%). Further, 52.4% of older brothers lived at home, 82.1% of younger brothers lived at home, 34.1% of older sisters lived at home, and 71.9% of younger sisters lived at home.

### Is There an Association between Caregiving, Birth Order, and Gender?

Scores on the ECI according to sibling gender and birth order are presented in **Table 3**. A series of one-way between groups ANOVA were performed to explore the difference between sibling groups and the Negative Aspects of Caregiving Subscale. Older brothers scored significantly lower burden scores than younger sisters overall [*F*(3,153) = 2.94, *p* = 0.035] (eta squared was 0.05). Younger sisters reported the highest burden for six of the eight items (stigma, problems with services, the effects on family, needing to provide back up, dependency and loss). There was a statistically significant difference for the experience of difficult behaviors between older brothers and older sisters with older brothers scoring lower [*F*(3,153) = 3.20, *p* = 0.025] (eta squared 0.06). Younger brothers reported a significantly lower score on the dependency subscale than younger sisters [*F*(3,153) = 3.74, *p* = 0.012] (eta squared 0.07). Regression analysis showed that a significant predictor of high burden was female gender. Regression analysis also showed that living at home did not explain the burden difference between older brothers and younger sisters.

**Table 3:** The experience of caregiving, sibling gender, and birth order.

	OB	YB	OS	YS	Total
	*N* = 42	*N* = 39	*N* = 44	*N* = 32	*N* = 157
*Mean (SD)*					
Difficult behaviors	12.73 (5.76)	16.84 (8.30)	16.95 (7.56)	16.75 (7.82)	15.75 (7.53)
Negative symptoms	12.61 (6.40)	12.23 (5.91)	15.25 (6.17)	14.31 (6.80)	13.60 (6.37)
Stigma	10.07 (4.59)	11.76 (5.60)	10.97 (3.37)	12.28 (5.35)	11.19 (4.76)
Problems with services	10.02 (3.71)	10.30 (3.89)	12.22 (5.77)	12.46 (4.99)	11.21 (4.76)
Effects on family	11.54 (5.09)	12.25 (5.67)	13.04 (4.87)	14.34 (6.65)	12.71 (5.56)
Need to back up	9.16 (3.58)	9.25 (3.10)	10.88 (4.42)	11.25 (5.03)	10.09 (4.12)
Dependency	8.59 (3.60)	7.94 (3.74)	10.18 (4.64)	10.68 (4.26)	9.30 (4.19)
Loss	12.90 (6.20)	12.76 (5.17)	13.75 (4.89)	16.37 (7.19)	13.81 (5.94)
Positive experiences	13.21 (6.34)	12.69 (6.03)	16.68 (7.04)	17.56 (6.77)	14.94 (6.82)
Good aspects	14.88 (5.32)	14.38 (4.78)	15.72 (4.86)	18.06 (5.43)	15.64 (5.21)
Total negative score	87.62 (4.70)	93.30 (5.30)	103.24 (4.86)	108.4 (6.10)	97.66 (5.30)
Total positive score	28.09 (1.55)	27.07 (1.30)	32.40 (1.45)	35.6 (1.53)	30.58 (1.52)

*Results of the Experience of Caregiving Inventory (ECI). Scores are presented for sibling gender and position [older brother (OB), younger brother (YB), older sister (OS), younger sister (YS)], and in total numbers [total siblings (T)].*

A series of one-way between groups ANOVA were performed to explore the associations between sibling groups for the Positive Aspects of Caregiving Subscales of the ECI. A statistically significant difference was found between the four dyads with brothers scoring significantly lower than younger sisters [*F*(3,153) = 5.25, *p* = 0.002] (eta squared was 0.09). Younger brothers reported the lowest score. Younger sisters reported the highest score. Statistically significant differences were found on both items of the Positive Aspects of Caregiving Scale: positive personal experiences [*F*(3,153) = 5.22, *p* = 0.002] (eta squared was 0.09) and good aspects of the relationship [*F*(3,153) = 3.52, *p* = 0.017] (eta squared was 0.06) with both older and younger brothers scoring significantly lower than younger sisters.

In summary, younger sisters experienced more burden than other sibling groups and they also experienced the most positive experiences. Sisters experienced the most overall burden.

### Caregiving and the Impact on Sibling Relationship

#### Is There an Association between Caregiving and the Sibling Relationship?

##### Warmth

A negative correlation was found between the Negative Aspects of Caregiving Subscale and warmth (*r* = 0.437, *p* < 0.001) with high scores on the Negative Aspects Subscale associated with less warmth within the sibling relationship (see **Table 4**). The eight items of the Negative Aspects Scale were analyzed separately to ascertain whether specific negative experiences were associated warmth within the relationship. Negative correlations were found between difficult behaviors (*r* = 0.631, *p* < 0.001), negative symptoms (*r* = 0.51, *p* < 0.001), stigma (*r* = 0.42, *p* < 0.001), perceived effects on the family (*r* = 0.39, *p* < 0.001) and warmth. A positive correlation was found for good aspects in the relationship subscale (*r* = 0.54, *p* < 0.001) and warmth.

**Table 4:** The experience of caregiving and the sibling relationship.

	Warmth		Conflict		Rivalry	
	*r*	*p*	*r*	*p*	*r*	*p*
Difficult behaviors	-0.63	0.001	0.62	0.001	0.56	0.001
Negative symptoms	-0.51	0.001	0.41	0.001	0.40	0.001
Stigma	-0.42	0.001	0.35	0.001	0.41	0.001
Problems with services	-0.14	0.700	0.57	0.001	0.21	0.008
Effects on family	-0.39	0.001	0.56	0.001	0.48	0.001
Need to back up	-0.19	0.120	0.53	0.001	0.28	0.001
Dependency	-0.39	0.623	0.45	0.001	0.23	0.003
Loss	-0.24	0.002	0.33	0.001	0.37	0.001
Positive personal experiences	0.72	0.371	0.51	0.001	0.11	0.171
Good aspects of relationship	0.54	0.001	0.05	0.485	-0.23	0.003
Total negative score	-0.43	0.000	0.60	0.000	-0.48	0.000
Total positive score	0.34	0.000	0.31	0.000	-0.07	0.355

*The 10 subscales of The Experience of Caregiving Inventory (ECI) and The Adult Sibling Relationship Questionnaire (ASRQ).*

##### Conflict

A positive correlation was found between Negative Aspects of Caregiving and conflict (*r* = 0.6, *p* < 0.001). Strong positive correlations were found between difficult behaviors (*r* = 0.626, *p* < 0.001), problem with services (*r* = 0.571, *p* < 0.001), effects on the family (*r* = 0.568, *p* < 0.001) and conflict.

##### Rivalry

A positive correlation was found between Negative Aspects of Caregiving and rivalry (*r* = 0.49, *p* < 0.001).

In summary, high scores on the Negative Caregiving subscale was associated with less warmth, more conflict and rivalry within the sibling relationship. Good aspects of caregiving were associated with more warmth in the relationship.

### Caregiving and the Sibling Quality of Life

#### Is There an Association between Caregiving and the Sibling Quality of Life?

##### Physical domain

In the physical domain (*r* = -0.477, *p* < 0.001), negative correlations were found between difficult behaviors (*r* = -0.427, *p* < 0.001), negative symptoms (*r* = -0.385, *p* < 0.001), problems with services (*r* = -0.364, *p* < 0.001), effects on family (*r* = -0.375, *p* = 0.001), need to provide back up (*r* = -0.382, *p* < 0.001), dependency (*r* = -0.444, *p* = 0.001), loss (*r* = -0.352, *p* < 0.001) and the physical domain (see **Table 5**).

**Table 5:** The experience of caregiving and the sibling quality of life.

	Physical domain		Psychological domain		Social domain		Environment domain	
	*r*	*p*	*r*	*p*	*r*	*p*	*r*	*p*
Difficult behaviors	-0.42	0.001	-0.38	0.001	-0.51	0.001	-0.45	0.001
Negative symptoms	-0.38	0.001	-0.36	0.001	-0.39	0.001	-0.39	0.001
Stigma	-0.27	0.001	-0.25	0.001	-0.45	0.001	-0.33	0.001
Problems with services	-0.36	0.001	-0.04	0.614	-0.19	0.016	-0.38	0.001
Effects on family	-0.37	0.001	-0.28	0.001	-0.43	0.001	-0.44	0.001
Need to back up	-0.38	0.001	-0.17	0.027	-0.29	0.001	-0.38	0.001
Dependency	-0.44	0.001	-0.22	0.005	-0.32	0.001	-0.43	0.001
Loss	-0.35	0.001	-0.24	0.002	-0.34	0.002	-0.36	0.001
Positive personal experiences	-0.32	0.001	-0.09	0.216	-0.12	0.118	-0.33	0.001
Good aspects of relationship	0.00	0.943	0.15	0.058	0.20	0.008	-0.03	0.680
Total negative score	-0.47	0.000	-0.33	0.000	-0.48	0.000	-0.50	0.000
Total positive score	-0.17	0.026	0.03	0.701	0.04	0.540	-0.17	0.03

*The 10 subscales of The Experience of Caregiving Inventory (ECI) and WHOQoL-BREF.*

##### Psychological domain

In the psychological domain (*r* = -0.334, *p* < 0.001), negative correlations existed between difficult behaviors (*r* = -0.382, *p* < 0.001), negative symptoms (*r* = -0.367, *p* < 0.001), stigma (*r* = -0.258,), effects on family (*r* = -0.288, *p* < 0.001), need to back up (*r* = -0.177, *p* < 0.001), dependency (*r* = -0.223, *p* < 0.001), and loss (*r* = -0.244, *p* < 0.001) and the psychological domain of quality of life.

##### Social domain

In the social domain (*r* = -0.486, *p* < 0.001), negative correlations existed between difficult behaviors (*r* = -0.512, *p* < 0.001), (*r* = -0.397, *p* < 0.001), stigma (*r* = -0.456, *p* < 0.001), effects on family (*r* = -0.439, *p* < 0.001), dependency (*r* = -0.223, *p* < 0.001), and loss (*r* = -0.244, *p* < 0.001) and the social domain.

##### Environment domain

In the environment domain (*r* = -0.508, *p* < 0.001), negative correlations existed between all subscales of the ECI and the environment domain of quality of life.

In summary, a negative correlation was found between Negative Aspects of Caregiving Subscale and all domains of the siblings’ quality of life.

### Caregiving and Illness Related Variables of FEP

#### Is There an Association between Caregiving and the Illness Related Variables Associated with FEP?

Independent-samples *t*-tests showed significant associations between the ECI Negative Aspects of Caregiving and attempted suicide [*t*(155) = -4.65, *p* < 0.001]; a history of violence [*t*(155) = 4.75, *p* < 0.001, η^2^ = 0.12]; persisting psychotic symptoms [*t*(155) = 6.07, *p* < 0.001, η^2^ = 0.19]; and persisting drug use [*t*(155) = 2.90, *p* = 0.004, η^2^ = 0.05] (see **Table 6**). ANOVAs showed that having more than one admission to hospital was associated with a high score on the Negative Aspects of Caregiving subscale, [*F*(3,155) = 4.5, *p* = 0.005] (see **Table 6**). Regression analysis showed that significant predictors of high burden were suicide attempts and a history of violence (see **Table 7**).

**Table 6:** The experience of caregiving and illness related variables of FEP.

Negative appraisal	Mean (SD)	(95%CI)	*p*	η^2^
Suicide attempts			0.000	0.063
Yes *N* = 45	18.12 (5.18)			
No *N* = 112	14.04 (4.88)			
Persisting psychosis			0.000	0.192
Yes *N* = 88	17.26 (4.72)			
No *N* = 69	12.59 (4.82)			
Duration of untreated psychosis (months)			0.802	0.002
1–6 *N* = 42	14.75 (5.94)	12.90–16.60		
7–12 *N* = 61	15.45 (4.96)	14.18–16.72		
>13 *N* = 54	15.29 (5.19)	13.87–16.71		
History of violence			0.000	0.120
Yes *N* = 15	21.00 (4.11)			
No *N* = 142	14.59 (5.013)			
Compliance			0.103	0.021
Yes *N* = 124	14.85 (5.41)			
No *N* = 33	16.54 (4.64)			
Persisting drug use			0.004	0.051
Yes *N* = 42	17.19 (5.60)			
No *N* = 115	14.48 (5.00)			
Living with ill brother or sister			0.532	0.008
Yes *N* = 92	14.99 (5.86)			
No *N* = 65	15.51 (4.37)			
Number of admissions			0.005	0.081
0 *N* = 41	14.56 (6.00)	12.67–16.46		
1 *N* = 49	13.53 (5.32)	12.00–15.06		
2 *N* = 36	16.24 (4.27)	14.79–17.69		
>3 *N* = 31	17.50 (4.35)	15.90–19.10		

*The impact of the characteristics of FEP upon the Negative Aspects of Caregiving (ECI).*

**Table 7:** Standardized (β) and unstandardized (*B*) regression coefficients from two standard regression analyses with the total negative and positive subscales serving as dependent variables in each model and the predictors including suicide attempt, history of violence, number of admissions, substance use, age and gender.

	Negative Aspects of Caregiving				Positive Aspects of Caregiving Subscale			
	β	*B*	*SE*	*p*	β	*B*	*SE*	*p*
Suicide attempts	0.269	20.227	5.86	0.001	0.178	4.194	2.05	0.043
History of violence	-0.300	-34.728	8.51	0.000	0.002	0.056	2.99	0.985
Number of admissions	0.072	2.280	2.50	0.363	-0.062	-0.613	0.87	0.487
Persisting substance use	-0.049	-3.750	6.13	0.542	0.156	3.748	2.15	0.084
Age	-0.084	-0.655	0.61	0.290	0.053	0.129	0.19	0.495
Gender	0.198	13.466	5.39	0.014	0.273	5.813	1.65	0.001
Lives with ill brother or sister	-0.001	-0.009	0.01	0.919	0.049	0.150	0.03	0.084

**Table 8:** Cronbach alpha coefficient for the Experience of Caregiving Inventory (ECI) (Szmukler et al., 1996), the Adult Sibling Relationship Questionnaire (ASRQ) (Stocker et al., 1997).

Experience of caregiving items	Study sample	Szmukler et al., 1996
	α	α
Difficult behaviors	0.91	0.91
Negative symptoms	0.91	0.89
Stigma	0.87	0.82
Problems with services	0.88	0.90
Effects on family	0.82	0.82
Need to back up	0.72	0.76
Dependency	0.80	0.74
Loss	0.85	0.79
Positive personal experiences	0.88	0.86
Good aspects of the relationship	0.83	0.82

**Sibling relationship items**	**Study sample**	**Stocker et al., 1997**
	**α**	**α**

Warmth	0.98	0.97
Acceptance	0.93	0.88
Admiration	0.94	0.83
Affection	0.94	0.92
Emotional support	0.94	0.90
Intimacy	0.95	0.92
Instrumental support	0.82	0.76
Knowledge	0.90	0.88
Similarity	0.90	0.83
Conflict	0.92	0.93
Antagonism	0.88	0.90
Competition	0.72	0.85
Dominance	0.79	0.74
Quarreling	0.87	0.86
Rivalry	0.91	0.88
Maternal rivalry	0.92	0.85
Paternal rivalry	0.95	0.89

**WHOQol- Bref domains**	**Study sample**	**World Health Organisation [WHO], 1998**
	α	α

Physical domain	0.70	0.68–0.74
Psychological domain	0.79	0.79–0.80
Social domain	0.79	0.68–0.70
Environment domain	0.79	0.84–0.87

### Summary of Results

Younger sisters experienced both the most burden and the most positive experiences. Younger brothers experienced the least positive experiences. A significant predictor of high burden was female gender. Living at home did not explain the burden difference between brothers and sisters. High burden was associated with less warmth, more conflict and rivalry within the sibling relationship. Good aspects of caregiving were associated with more warmth. High burden was associated with less satisfaction in all domains of siblings’ quality of life. Significant predictors of high burden were suicide attempts and a history of violence.

## Discussion

This research found through univariate and multivariate analyses that there were two illness-related variables of FEP that resulted in high levels of burden for siblings. If the young person experiencing FEP had attempted suicide or had a history of violence, then these factors were significant predictors of the sibling appraising their caregiving experiences negatively. Females, particularly younger sisters, experienced high levels of caregiver burden. They also reported the most positive experiences. These findings are discussed in the sections that follow.

### Suicide Attempts

Suicide is a leading cause of premature death in FEP populations (Nordentoft et al., 2011). Up to 46% of families in FEP will have experienced their child/ brother or sister expressing suicidal ideation; 18% will have experienced a suicide attempt prior to receiving treatment; and 20% will have experienced a suicide attempt after treatment has commenced (Robinson et al., 2009; Challis et al., 2013). Research shows that the majority of individuals experiencing FEP who attempted suicide live with their families and suicidal behaviors take place at home (Nordentoft et al., 2011, 2013; Large et al., 2014). In most instances, a family member discovers the person during or after the attempt (Fedyszyn et al., 2014). Siblings have been found in research to have unique knowledge of their brother or sister’s suicide attempts, the triggers, and the causes (Dyregrov and Dyregrov, 2005).

In an Australian study with siblings of young people experiencing FEP, Bowman et al. (2014) found that suicide attempts resulted in less satisfaction in the siblings’ quality of life. Sisters were more vulnerable to the effects of suicide with significant impact made to their quality of life. Suicide attempts in FEP have also been found to impact upon the sibling relationship by reducing warmth and increasing conflict (Bowman et al., 2015). This consequently may have an impact on the protective and supportive qualities within the sibling relationships. It is therefore not surprising that in this study an association was found between suicide attempts and high scores on the negative experience of caregiving. These results add further depth to the impact that suicide attempts can have on the sibling by showing its association with high levels of burden which has an impact on psychological well-being.

Studies have demonstrated that a long duration of untreated psychosis (DUP) is a high risk period for suicide and suicide attempts (Barrett et al., 2010; Falcone et al., 2010). Studies have also found intensive early detection programs are effective and can significantly shorten the DUP (Joa et al., 2008). It has been shown that an early detection program can assist young people before complications of untreated illness such as suicidal plans and acts have developed (Melle et al., 2004). There is evidence that DUP is related to higher burden of care for families so intervention efforts should focus on minimizing it Alvarez-Jimenez et al. (2010). It is suggested that ongoing research and clinical efforts to reduce the DUP would also assist siblings by reducing their burden of care. This could be achieved by increasing public awareness of the early warning signs and symptoms of FEP in schools and general practitioner clinics. Further, ensuring through public awareness that siblings know where to get help and how to support their brother or sister in facilitating clinical assistance as soon as possible.

In a Norwegian mixed method study Dyregrov and Dyregrov (2005) (*n* = 70) explored the needs of siblings after losing a brother or sister to suicide. The first subsample consisted of 11 adolescents (five younger sisters, six younger brothers) with a mean age of 17.7 years who lived at home with their parents at the time of the death. The second subsample consisted of 59 siblings (39 older sisters, 20 older brothers) with a mean age of 28.4 years who lived either alone or with their own core family at the time of the death.

One-third of younger siblings had been aware of previous suicide attempts, knew the triggers and did not tell their parents which burdened them with guilt. Whilst the parents reported finding it difficult to understand why the suicide had happened, the siblings had their own theories as to why it happened. Siblings reported they did not talk about this with their parents and they did not communicate their own grief. As a result they felt alone. Siblings did not feel looked after by the family’s network and professionals because most of the attention was directed toward their parents. The parents also confirmed that the bereaved siblings were “forgotten” in the days following the death. There is evidence that siblings have largely been forgotten in research as well as clinical practice (Bowman et al., 2013).

Qualitative studies have found that siblings in FEP provide support to their parents rather than the other way around and see this as one of their roles (Newman et al., 2011; Sin et al., 2011). Parents may be less available for the sibling because of the stress and burden they themselves experience. As well as enhancing risk recognition through psychoeducation with families, we suggest that providing education to parents about the impact of suicide attempts on the sibling relationship, their quality of life and the burden they experience, should be included and become regular practice by clinicians in FEP services. The priority should however, be to engage and support siblings in their own right.

Promoting positive caregiving experiences for siblings may promote warmth and the reciprocal protective benefits within the relationships. Interventions that support the importance of the sibling relationship during critical incidents such as suicide attempts would be beneficial not only to the psychological well-being of the sibling but also in managing risk due to the intimate knowledge the siblings can have of each other (Dyregrov and Dyregrov, 2005).

Cerel et al. (2008) suggested that suicide attempts have a profound effect on social networks for family members. Family members can blame themselves for words that were exchanged with the ill individual, or for their seeming shortcomings as a parent, partner, sibling, or so forth. Even if they conclude that they were not directly responsible for the attempt, family members can struggle with the perceived failure to anticipate and intervene to prevent it from occurring. Younger sisters in this study reported the most burden and may be particularly sensitive to suicide attempts.

Qualitative studies have shown that siblings of individuals with long term psychosis want effective and assertive interventions for their ill brother or sister (Riebschleger, 1991; Gerace et al., 1993). We suggest that young people who are identified at high risk of suicide by clinicians be allocated more intensive clinical intervention support. Intensive clinical support reduces suicide risk (Brewer et al., 2015). This may reduce the burden experienced by siblings and promote positive caregiving within the relationship. For example, after an investigation into completed suicides in the EPPIC program, an intensive case management subprogram was developed to provide assertive outreach to young people having a FEP who were identified by their clinical team as being at high risk to self or others, of disengagement, or prolonged recovery. Brewer et al. (2015) evaluated this subprogram and found significant improvement in clinical outcomes. No suicides occurred at EPPIC during the life of this subprogram (Brewer et al., 2015). Targeting intensive case management services to high risk individuals with unmet needs can reduce risks, facilitate recovery and the burden experienced by family members including siblings (Brewer et al., 2015).

### History of Violence

It has been found in studies from the United Kingdom, Ireland, Canada, and Australia that up to 42% of young people with FEP commit an act of physical violence (Milton et al., 2001; Foley et al., 2007; Harris et al., 2008; Spidel et al., 2010; Large and Neilssen, 2011). Studies have shown that even family members who do not live with the ill individual still have significantly high psychological impact from the trauma of the individual’s behavior (Hanzawa et al., 2013). In studies with individuals experiencing long term psychosis, siblings are the second most common targets of physical violence after violence against parents (Solomon et al., 2005).

A history of violence by the young person with FEP has been found to have a significant and negative impact on all domains of quality of life for siblings in FEP and a damaging impact on the sibling relationship (Bowman et al., 2014, 2015). A history of violence was found to be associated with less warmth and more conflict within the relationship. This study has added further insight into the experience of siblings, showing that it also results in significant burden that has implications for psychological well-being. The results of this study are consistent with those about siblings in long-term psychotic illness as burden is associated with physical violence for siblings of adults with long-term psychotic illness (Greenberg et al., 1997). This study has found that burden exists for siblings even during the first experience of a young person’s psychosis (Solomon et al., 2005; Smith and Greenberg, 2008).

This study has found that a history of violence results in high burden for siblings in FEP. Again, it is therefore suggested that young people with FEP who are identified as high risk of violence by clinicians, be allocated more intensive clinical support. Physical violence is often associated with persisting psychotic symptoms, substance use and non-compliance with treatment (Brewer et al., 2015). In a naturalistic stratified quasi-experimental real-world design by Brewer et al. (2015) many young people in their sample had a forensic history (as indicated by a formal police record) and a history of violence toward others. As a result of intensive case management, there were significant reductions in hospitalisations and crisis contacts, improvements in symptoms and global functioning. There were also no adverse events and risk ratings reduced significantly. Furthermore, there were no failed referrals or treatment dropouts in this difficult-to-engage cohort, validating the intensive case management model of engagement. The evidence shows that this model reduces the risk of violence in this high risk group which has implications for the sibling experience.

It is suggested that suicide attempts and a history of violence are indicators for more intensive case management services as described by Brewer et al. (2015). This could be a way to reduce the burden experienced by siblings and further support the sibling relationship and the social support it inherently provides thus promoting recovery for the individual with FEP. Further research into this intensive model of case management and its impact on the experience of caregiving for siblings is indicated. When a suicide attempt occurs for a young person with FEP, or when an episode of violence has occurred, clinicians should be assertive in contacting the sibling and offering debriefing, support and psychological interventions if required. Siblings may also require practical support regarding the impact it may have on their daily functioning and quality of life. The inclusion of siblings in family interventions should become routine practice. If clinicians and research continue to neglect the inclusion of siblings in FEP, loss of this relationship may result in a significant loss of social support and all the protective benefits this relationship can possess (Branje et al., 2004; Milevsky, 2005; Gass et al., 2007). Research shows that social support promotes recovery in FEP (Norman et al., 2005; SANE Australia, 2010). Maintaining these protective benefits may also reduce the negative impact upon the sibling in accordance with positive psychology (Seligman and Csikszentmihalyi, 2000; Bowman et al., 2013, 2014).

### Gender and Birth Order

The findings of this study support previous gender research where females reported more negative experiences than men (Fujita et al., 1991; Polce-Lynch et al., 2001; Moksnes et al., 2010). Researchers have suggested that males and females are equal in their experiences but men are more reluctant than women to admit negative experiences and feelings (Fujita et al., 1991; Ladwig et al., 2000; Moksnes et al., 2010). In this study, brothers reported the lowest burden and sisters reported the highest. It is possible that sisters have endorsed the items more strongly and given more extreme responses than brothers in this study giving the result of higher and negative caring experiences. These results may also be explained in light of theories of gender relationships.

In this study brothers reported 15 points less than older sisters and 20 points less than younger sisters on their total negative score. This is in agreement with studies about siblings in long term psychosis. For example, Barak and Solomon (2005) found that sisters reported greater burden than brothers. The authors found that sisters more than brothers served as sources of comfort and support for parents and their ill brother or sister which increased their experience of burden (Barak and Solomon, 2005).

Sin et al. (2011) in their qualitative study with siblings in FEP also found that brothers and sisters reported different caregiving experiences. Further Newman et al. (2011) explored the impact of the experience on a siblings’ sense of self and the roles they adopted within the family. The cross-case analysis indicated a gender difference for this experience. The emphasis for women was on finding personal meaning and for the men it was on taking up responsibilities both within the family and those that promoted individuation from the family.

In this study, older siblings reported lower burden than younger siblings. Most younger siblings lived at home. Consequently, younger siblings were exposed to more negative experiences associated with FEP and the characteristics of the illness. It is therefore not surprising, considering theories of gender relationships, that younger sisters reported the highest levels of burden from stigma, problems with services, the effects on family, needing to provide back up, dependency and loss. Younger sisters also scored highest on questions that asked about covering up their brother or sister’s illness, feeling unable to tell anyone and feeling unable to have visitors home. This may indicate that younger siblings are particularly vulnerable and have less social support than other sibling groups which may be critical due to the disruption and potential loss of the protective and supportive benefits of the sibling relationship. Younger sisters experienced the most burden but also reported a greater number of positive experiences than other sibling groups. Sisters living at home may feel more emotionally involved in the care of the young person with FEP hence the fact they experience both more negative and positive experiences of caregiving.

Stigma has also been identified in the literature as a significant burden for siblings in long term psychosis with younger siblings experiencing more stigma than older siblings (Greenberg et al., 1997). The current study supports these findings as the results show that stigma exists particularly for younger siblings in FEP. This has implications for psychoeducation.

The finding is again consistent with the research by Dyregrov and Dyregrov (2005), who reported that birth order was relevant to the level of distress experienced by siblings following suicide. Older siblings experienced less distress than the younger siblings due to their age and developmental stage (often living out of home), marital status and external social support. These factors may also protect older siblings of young people with FEP as they are more able to avoid exposure to their parent’s distress and the characteristics of FEP such as hospital admissions, non-compliance with treatment, and/or persisting symptoms. Being older may mean that the ill brother or sister is not a prominent source of social support or role model and therefore they may feel more detached or distant as they already have external support networks that are protective.

Due to the high level of burden reported by the participants in this study, a change in the protective effects of sibling support may exist and should be further investigated. When a young person is experiencing FEP, they may be unable to provide the previously experienced support and relationship to their sibling. This may result in increased distress for siblings when dealing with stressful life events due to the loss of comfort and security, resulting in changes to psychological adjustment, well-being, and self-esteem. This may contribute to their appraisal of caregiving in FEP. Further, previous research in FEP has shown that siblings are often forgotten and not included. This research shows that siblings are providing a great deal of care to their ill brother or sister and consequently reporting high levels of burden. The results of this research indicate that interventions should focus more strongly on younger siblings, foster warmth and reduce detachment in males, and reduce burden and potential emotional over-involvement of females.

### Limitations

Participants in this study were self-selected and therefore may represent a group of siblings who had experienced greater hardship. Consent was required from the young person experiencing FEP and this may have contributed to the omission of a specific group of siblings and selection bias. Data were not collected regarding family dynamics that may have contributed further insight into the caregiving activities of the sibling and the family factors that impacted upon it. It should be acknowledge that the ECI measure was not specifically developed to explore the siblings’ experience of caregiving but was developed with parents and their experiences in mind. It is therefore possible that unique sibling experiences may have been missed in this study such as fear of becoming unwell themselves. The cross-sectional nature of this study precludes conclusions on the directions of the associations.

### Clinical Implications

The findings of this research show that siblings in FEP experience significant burden due to their negative appraisal of caregiving. This is particularly high if the young person with FEP has attempted suicide and/or been physically violent. Further, younger sisters are particularly vulnerable to burden. Clinicians can use these findings to identify siblings and assertively intervene to provide increased psychological support, psychoeducation, and practical problem solving to reduce the burden. Clinicians should also be aware of the impact on siblings of high risk illness related variables such as suicide attempts and violence. These factors are indicators for intensive case management in order to improve outcomes for the individual with FEP which may in turn reduce the burden experienced by the sibling.

This research found that positive experiences of caregiving fostered warmth and negative experiences fostered conflict and rivalry within the sibling relationship. Strengthening the sibling relationship by promoting positive caregiving experiences could assist to preserve the quality of the relationship, promote healthy family functioning, foster psychological health and prevent risk taking behaviors in the individual with FEP.

This research also found that psychoeducation is indicated in reducing the stigma experienced by siblings in FEP as well as recognizing the caregiving role that they already play for their ill brother or sister. Parents should be educated about the importance of the sibling relationship and the impact the illness can have on the sibling’s quality of life and the burden they can experience. Siblings in FEP should no longer be forgotten.

## Author Contributions

SB wrote the initial draft of the paper. MA-J, DW, LH, PM reviewed the paper and suggested changes and amendments. SB integrated all recommendations into the paper. The paper was then resent to all authors for their review. All authors were happy with the final submission.

## Conflict of Interest Statement

The authors declare that the research was conducted in the absence of any commercial or financial relationships that could be construed as a potential conflict of interest.
